# Biologically Active Supplements Affecting Producer Microorganisms in Food Biotechnology: A Review

**DOI:** 10.3390/molecules28031413

**Published:** 2023-02-02

**Authors:** Artem P. Dysin, Anton R. Egorov, Anastasia A. Godzishevskaya, Anatoly A. Kirichuk, Alexander G. Tskhovrebov, Andreii S. Kritchenkov

**Affiliations:** 1 Faculty of Science, Peoples’ Friendship University of Russia (RUDN University), Miklukho-Maklaya St. 6, 117198 Moscow, Russia; 2 Metal Physics Laboratory, Institute of Technical Acoustics NAS of Belarus, Ludnikova Prosp. 13, 210009 Vitebsk, Belarus

**Keywords:** biotechnology, microorganisms, polysaccharides, oligosaccharides

## Abstract

Microorganisms, fermentation processes, and the resultant metabolic products are a key driving force in biotechnology and, in particular, in food biotechnology. The quantity and/or quality of final manufactured food products are directly related to the efficiency of the metabolic processes of producer microorganisms. Food BioTech companies are naturally interested in increasing the productivity of their biotechnological production lines. This could be achieved via either indirect or direct influence on the fundamental mechanisms governing biological processes occurring in microbial cells. This review considers an approach to improve the efficiency of producer microorganisms through the use of several types of substances or complexes affecting the metabolic processes of microbial producers that are of interest for food biotechnology, particularly fermented milk products. A classification of these supplements will be given, depending on their chemical nature (poly- and oligosaccharides; poly- and oligopeptides, individual amino acids; miscellaneous substances, including vitamins and other organic compounds, minerals, and multicomponent supplements), and the approved results of their application will be comprehensively surveyed.

## 1. Introduction

Modern biotechnology is rapidly expanding becoming more influential and complex as it gradually penetrates into all aspects of people’s everyday life. Back in 1970, this term was mainly referred to as food industry and agriculture. Currently, biotechnology includes such essential subareas as bioengineering, biomedicine, bioinformatics, bionanotechnology, bionics, and genetic engineering [1]. However, food biotechnology still takes a leading position, being replenished with new high-tech methods and approaches, resulting in the development of innovative tools such as probiotics, prebiotics, synbiotics, and other functional nutritional supplements.

Fermentation processes are at the heart of traditional biotechnology and the biotechnology of food, in particular. Throughout history, fermentation processes have been used to prepare a wide variety of everyday foods, such as cheese and yogurt, bread, and alcoholic beverages [2,3,4,5]. The microbial fermentation of food raw materials is a complex process, which results in the formation of a wide assortment of metabolites, which are responsible for the taste and flavor of the product on the one hand and the consumer’s health benefits on the other [6]. Although modern knowledge does not question whether microbial fermentation is driven by enzymes, microorganisms producing enzymes rather than enzymes themselves in their pure form are typically exploited in biotechnology.

Due to the ever-growing demand for biotechnological products, a significant intensification of enzymatic processes is urgently required. The current global economic situation requires biotechnology to meet the following challenges:

1. Increase the number of biotechnological products available on the market;
2. Save natural resources (raw materials and microbial biomass);
3. Reduce energy consumption, and the technological and labor power costs of production.

All these problems can be largely solved by intensifying microbial fermentation in food biotechnology. Consequently, the intensification of microbial fermentation processes is one of the most important tasks of modern biotechnology.

Another important aspect of the modern food industry, as well as medicine and pharmaceutics, is the stimulation and normalization of the activity of probiotic microorganisms in the human gastrointestinal tract. Probiotics are living microorganisms that provide health benefits by improving or restoring intestinal flora. Disruption of the activity of probiotic microorganisms causes severe pathologies (dysbiosis, etc.). Food, especially fermented milk products (yogurt, kefir, and cheese), is the most essential source of probiotic microorganisms, among which strains of the genera *Lactobacillus* and *Bifidobacterium* are best known [7]. In modern biotechnology, the production of functional food products containing stress-resistant, well-functioning microorganisms has become an industrial standard. The proper selection of probiotic microorganisms with such properties is an important task of modern microbiology and food science. The relevance of this field is clearly demonstrated by the constantly increasing number high-impact publications.

Currently, the major efforts of the scientific community are aimed at the following:

1. The intensification of microbial fermentation processes;
2. An increase in stress resistance and the viability of probiotic microorganisms introduced into the human body as functional nutrients.

In general, two approaches are used to solve these essentially similar problems.

The first one involves breeding and genetic engineering. It implies that the genome of the producer microorganism is intentionally modified to enable the desired properties, such as the more intense synthesis of necessary metabolites, increased viability, and resistance to environmental conditions. With the help of genetic engineering, a number of recombinant producer microorganisms were engineered, which yielded a significant intensification in microbial fermentation. These outstanding advancements are meticulously discussed in recent reviews and books [8,9]. Furthermore, genetic engineering and selection allow for the production of probiotic microorganisms with improved stress resistance as reviewed in [10,11].

However, genetic engineering and breeding are not the only plausible tools that can be used to increase the efficiency of microbial production in food biotechnology. Bacteria are known to need the minimum number of essential nutrients to support growth and proliferation: water, a carbon source, a nitrogen source, and several mineral salts [12]. Water plays a fundamental role in the dissolution of nutrients, their transport, and hydrolysis reactions. Carbon is the most abundant and most important element for bacteria. Bacteria need to produce carbon-containing compounds such as fats, proteins, carbohydrates, and nucleic acids, using either inorganic (carbon dioxide) or organic precursors (saccharides and alcohols) [13]. For the synthesis of peptides, bacteria need nitrogen. Nitrogen sources can also be in either organic (protein hydrolysates: proteose peptone or tryptone) or inorganic (nitrates) form [14]. To maintain metabolic reactions in a steady state, bacterial cells need mineral salts (containing both anions such as phosphates or sulfates and cations such as magnesium and calcium) [12,13]. The type of source strongly affects the efficiency of assimilation of these elements by bacterial cells. Consequently, the choice of the most suitable sources of essential chemical elements for producer microorganisms is crucial to efficient biotechnological production. Typically, these components are added to the nutrient substrate as supplements.

In this regard, the present paper surveys the types of supplements that promote the growth, survival, and productivity of microorganisms relevant to the food industry and biotechnology, in particular lactic acid and/or probiotic bacteria. These supplements can be classified into three main groups, depending on their chemical identity:

1. Poly- and oligosaccharides;
2. Poly- and oligopeptides, individual amino acids;
3. Miscellaneous substances, including vitamins and other organic compounds, minerals, and multicomponent supplements.

**Figure 1:**
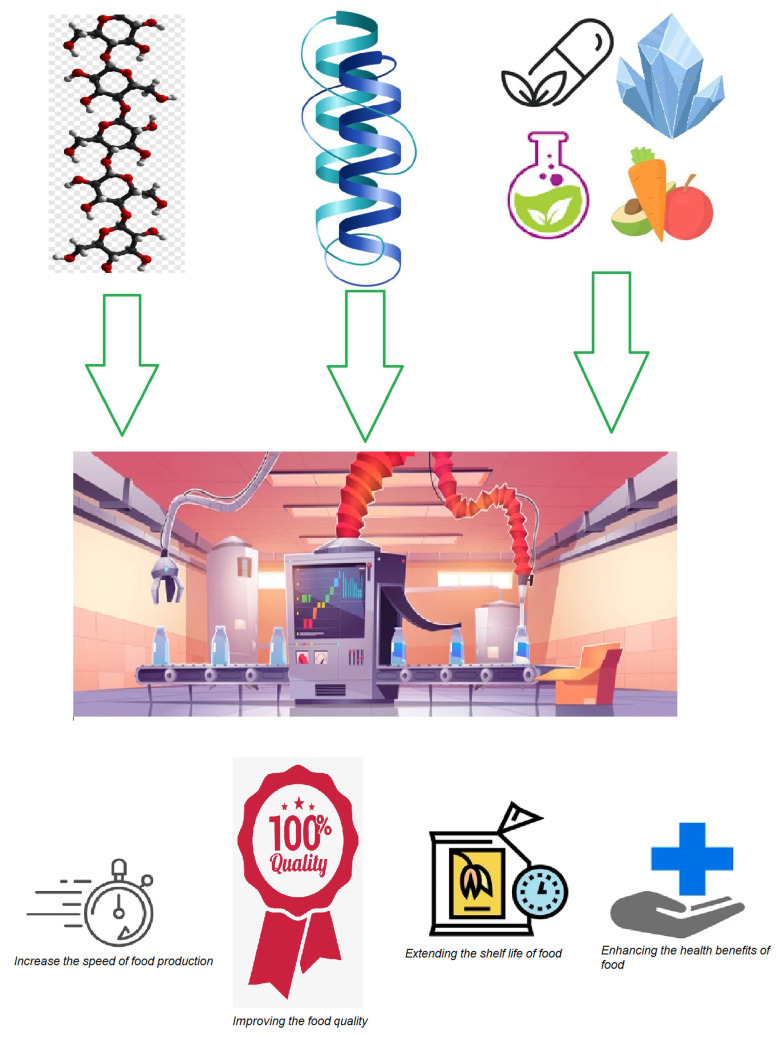
A schematic representation of the main idea behind this review.

A detailed description of the structure, biological effects, and potential applications of each type of supplement is given in the sections below. Figure 1 describes the overall essence of this review.

## 2. Poly-Oligosaccharide Supplements

Prebiotic carbohydrates are known to promote the growth and activity of beneficial microorganisms. These include fructo-, gluco-, galacto-, xylo-oligosaccharides, etc. They are obtained from monosaccharides or polysaccharides isolated from plants. Currently, fructo- and galacto-oligosaccharides are leaders in the world market of prebiotics [15]. Many strains of lactic acid bacteria have been found to grow better on fructo-, galacto-, and xylo-oligosaccharides than on corresponding monosaccharides as digesting substrates [16]. In this regard, oligosaccharides and dietary fiber are actively used in the development of fermented food products to enhance the properties of beneficial microorganisms, as well as their preservation in the product [17]. The combination of food additives consisting of probiotic microorganisms and prebiotics, called symbiotics, has shown promising effects in preventing disease and maintaining good health [18]. Within the context of the application of poly- and oligosaccharides as bioactive supplements, their effect on the growth, metabolism, and survival of bacteria directly participating in the fermentation stage of biotechnological product production should be described first. The use of these supplements as prebiotics to preserve probiotic bacteria in the active form is equally important. Table 1 lists the most notable of these supplements.

### 2.1. Fructans and Fructooligosaccharides

Inulin is one of the most studied and used prebiotic additives. It is a polysaccharide belonging to the class of fructans—fructose-derived polymers, mainly constructed from sucrose moieties linked by fructose units through β- (2 → 1) or β- (2 → 6) glycosidic bonds [19]. Currently, inulin is increasingly used in food, in particular in all types of dairy products, to promote beneficial bacterial activity [20]. Inulin and its oligosaccharides have been shown to exert a protective action on *Lactobacillus acidophilus* [21], *Lactobacillus casei* [22], *Lactobacillus paracasei* [23], *Lactobacillus rhamnosus* [24,25], *Streptococcus thermophiles* [25], *Lactobacillus plantarum* [26], and *Lactobacillus reuteri*, *Lactococcus,* and *Bifidobacterium* spp. [21,27,28], improving on their survival and post-storage activity [29]. Inulin has been reported to generate a more pronounced prebiotic effect than oligofructose, with respect to both enzymatic activity and the maintaining of the optimum composition of bacterial communities according to the simulation of the human intestinal microbial ecosystem [30]. Inulin is a fermentable fiber that cannot be cleaved by amylase or other hydrolytic enzymes in the upper intestinal tract. Like fructooligosaccharides, it is often used in studies in vivo, due to its resistivity to the action of the acidic environment typical of gastric and pancreatic enzymes [31].

In addition, inulin has proven to be a promising supplement that can enhance the biological activity of some microorganisms used in food enzymatic processes. The administration of inulin (as in the commercial product Raftiline HP^®^) significantly improved the growth of *S. thermophilus* as well as *Bifidobacterium longum* [28]. In the fermentation of kefir from soy milk, inulin promoted the survival of cells in the starter cultures of *Lactobacillus*, *Lactococcus*, and yeast [32]. Simultaneously, inulin had no effect on the survival rate and lactic acid production in starter cultures of *S. thermophilus* and *L. delbrueckii* subsp. *bulgaricus* during the storage of dry skimmed yogurt and *L. casei* [33].

It is also important to discuss the prebiotic properties of other fructans according to studies reported in the literature. The results show that branched fructans with a higher polymerization degree obtained from Aloe vera plants after water stress were more efficient than acemannan (acetylated glucomannan) and commercial fructooligosaccharides in stimulating the growth of various *Lactobacillus* species (*L. plantarum*, *L. casei*, *Lactobacillus fermentum*) and *Bifidobacterium* spp. (*B. catenalatum*, *B. bifidum*, *B. longum*, *B. animalis* spp. *lactis*) [34]. The *Agave salmiana* plant was also found to be a promising source of potential prebiotic fructans, which demonstrated an increased growth stimulation of *L. acidophilus* and *B. lactis* cultures in comparison with commercial fructan products [35]. The fructans of *A. salmiana* showed a prebiotic effect toward *L. casei* and *B. lactis*, comparable to that of inulin, and participation in the activation and selective differentiation of immune system cells through the interaction with probiotic microorganisms [36]. The prebiotic effect of fructans isolated from *Agave angustifolia* has also been studied depending on their polymerization degree. As it turned out, some *L. casei* subsp. *rhamnosus* and *L. plantarum* cannot use fructans as the only carbon source, regardless of their polymerization degree, while *Bifidobacterium* spp. grows only in the presence of low-molecular-weight fructans. However, certain strains of *B. adolescentis* and *B. infantis* were able to grow in media containing fructans of any molecular weight, and strains of *L. paracasei* subsp. *paracasei* and *B. bifidum* grow identically regardless of the carbon source of the fructan fraction [37]. Thus, it can be concluded that fructans with shorter molecules are preferable for the growth of probiotic microorganisms, which contradicts the observations by Quezada et al. (2017) [34] quoted above.

Fructooligosaccharides, which are essentially products of fructan hydrolysis, are oligosaccharides consisting of D-fructose units assembled via β-(2-1)-links. They are also not hydrolyzed by human digestive enzymes and may naturally occur in various plants such as leeks, garlic, asparagus, artichokes, dahlias, yacons, and chicory [38]. The supplementation of both fructooligosaccharides and inulin to yogurt almost equally caused an increase in the amount of the probiotics *S. thermophilus*, *L. acidophilus*, and *Bifidobacterium* sp. [39]. As for the metabolism of these bacteria, fermented soy milk supplemented with fructooligosaccharides showed a better acidification and post-acidification profile compared to samples supplemented with inulin, which significantly improved the physicochemical and sensory characteristics of the final product [40]. Additionally, fructooligosaccharides promoted the production of folic acid in five probiotic strains, *S. thermophilus* TH-4, *L. acidophilus* LA-5, *L. rhamnosus* LGG, *L. fermentum* PCC, and *L. reuteri* RC-14, added to soy milk and supported their survival [41,42]. Investigating the probiotic potential of the *L. plantarum* strain, Wang et al. (2019) [43] observed that different combinations of prebiotic oligosaccharides affect bacteria in different ways. For example, the combination of fructooligosaccharides and galactooligosaccharides significantly improved the viability of *L. plantarum* ZLP001 under heat stress, while fructooligosaccharides and soy oligosaccharides significantly increased microbial viability in response to cold stress [43].

At the same time, fructooligosaccharides, like mannan oligosaccharides, had no effect on the growth of *Enterococcus faecium*, which is one of the most common probiotics and microorganisms used in milk fermentation. However, when combined with gum arabic in the commercial product Preplex^®^, *E. faecium* NCIMB 30183 grew significantly faster [44]. Additionally, unlike inulin, fructooligosaccharides demonstrated no significant effect on the commercial *L. casei* strain added to a tropical fruit drink [24], and even caused a decrease in the biomass production of two *Lactobacillus plantarum* strains [45]. Among the selected 18 strains of *L. delbrueckii* subsp. *bulgaricus* and *S. thermophilus*, only two strains of lactobacilli were able to grow on media with fructooligosaccharides as a carbon source [46]. At the same time, compared to inulin, oligofructose significantly improved the viability of *L. acidophilus* La-5 and *B. animalis* Bb-12 in an ice cream mixture during freezer storage [47].

### 2.2. Lactulose and Galactooligosaccharides

The effect of another prebiotic, lactulose disaccharide synthesized from galactose and fructose, on bacterial growth was established mainly for Bifidobacteria (bifidogenic effect) [29], while for probiotic lactobacilli the effect of lactulose was less pronounced [48,49]. Moreover, supplementation with lactulose not only increased the viable cell count of *B. animalis* subsp. *lactis* and *B. longum* significantly, but also enhanced their promoted biotransformation of isoflavone glycosides to isoflavone aglycones in soy milk [50]. However, in an earlier study, the same authors found a similar effect of this disaccharide on some strains of lactobacilli [51].

The growth of lactobacilli being more strongly stimulated by the commercially available products Frutafit and Oligomate 55 (consisting mainly of inulin and galactooligosaccharides, respectively) than by lactulose has been detected [49]. However, another study demonstrated that lactulose had a significantly greater effect on the growth and enzymatic processes of *S. thermophilus* and *L. delbrueckii* ssp. *bulgaricus* in the production of yogurts than fructo- and galactooligosaccharides [52], as well as in the production of a fermented milk beverages [53]. The influence of glycosidic bonds in galactotrisaccharides synthesized from lactulose and lactose on the growth of *Lactobacillus*, *Streptococcus,* and *Bifidobacterium* was also established. The increased bacterial growth is possibly promoted by the specific monosaccharide composition of the galactooligosaccharides rather than additional glycosidic bonds [54].

As for the use of these oligosaccharides in the production of fermented products, neither lactulose, nor its derived galactooligosaccharides, nor commercial galactooligosaccharides from lactose affected the viability of lactic acid bacteria and yeast. Furthermore, they did not affect the concentrations of glycerol, lactic acid, pH, or short-chain fatty acids in kefir production [55]. However, an earlier study suggested that galactan oligosaccharides stimulated the growth of *L. delbrueckii* subsp. *bulgaricus* and *S. thermophiles* (10 out of 18) strains to a larger extent than fructo- and gluco-oligosaccharides [46].

### 2.3. Xylan and Xylooligosaccharides

Xylooligosaccharides are products of xylan hydrolysis containing oligomers of β-1,4-linked xylose residues with various substituents, including acetyl groups, phenols, and uronic acids [56]. They are usually obtained from oat xylan, birch wood, or corn cobs via enzymatic hydrolysis [57]. Xylooligosaccharides, along with mannanoligosaccharides, fructooligosaccharides, and chitosan oligosaccharides, were found to increase the tolerance of *L. plantarum* and *L. acidophilus* to heat, exposure to phenol solutions, and artificial gastrointestinal juices [58]. In a number of other studies, xylan oligosaccharides, like the previously described lactulose, were found to create a predominantly bifidogenic effect and exert a significantly lower influence on *Lactobacillus* [59,60,61,62]. 

Arabinoxylans, which are copolymers of arabinose and xylose, have also been successfully tested as potential prebiotics. Arabinoxylans from whole wheat flour were found to support the selective growth of *Bifidobacterium breve* and *L. reuteri* probiotics *in vitro*, providing a higher prebiotic activity than inulin [63], as well as the growth of *B. animalis lactis* in vivo [64].

At the same time, as in the case of inulin, supplementation with xylooligosaccharides did not affect the properties of yogurt starter cultures during storage [33]. According to another study, a prebiotic dose-dependent effect was observed when soy sauce sediment was added to the nutrient substrate, 53.2% of the molar fraction of which was xylooligosaccharides, towards *L. bulgaricus* and *S. thermophilus* used both as starter cultures for the fermentation of fermented foods and as probiotic additives [65].

### 2.4. Glucans and Glucooligosaccharides

(1,3)-β-glucans are widely considered as biological response modifiers. They attract the attention of the pharmaceutics and functional food industries due to their beneficial effects on human and animal health. Their biological effects are mainly influenced by their branching degree, molecular weight, and tertiary structure [66].

The supplementation of oat β-glucan to yogurt resulted in the improved viability and stability of *Bifidobacterium animalis* ssp. *lactis* comparable to those of inulin. It also increased the production of lactic and propionic acids [62]. Beta-glucan also generated a protective effect on strains of bifidobacteria in yogurt under stress during a prolonged refrigeration. The inclusion of beta-glucan in yogurt improved the survival of *B. longum* when stored at 4 °C [67]. The choice of the bifidobacteria strain, as well as the method for obtaining beta-glucan, proved to be vital for increasing the survival of the probiotic. For example, in the case of *B. longum*, the supplementation with beta-glucan increased the likelihood of the physiologically beneficial delivery of the probiotic even after 3 weeks of storage at 4 °C. At the same time, the starch taken for comparison had a positive effect on the survival of *S. thermophilus* and *L. bulgaricus*, but did not affect the survival of selected strains of bifidobacteria [68]. Russo et al. [60] managed to obtain 2-substituted (1-3)-β-d-glucan of bacterial origin, which promoted the growth of not only bifidobacteria, but also *L. plantarum* and *L. acidophilus* [59].

Pullulan is one of the best-known representatives of glucans, namely α-glucans. Recent studies have shown its growth-stimulating and protective effect on starter and probiotic lactobacilli and the bifidobacteria of yogurt, as well as a positive effect on their enzymatic characteristics related to yogurt acidity [69,70].

The prebiotic and synbiotic properties of oligosaccharides derived from β-glucans were also studied. For example, β-glucooligosaccharides derived from barley β-glucan, consisting of 3-O-cellobiosyl-d-glucose and 3-O-cellotriosyl-d-glucose, has recently been found to regulate selective growth and exert antimicrobial activity on *L. lactis* subsp. *lactis*, *L. reuteri*, and *Pediococcus acidilactici*, leading to an increase in their production of nisin Z [71]. According to Ignatova et al. (2009) [46], glucooligosaccharides had a positive effect on the growth of 6 out of 18 tested bacteria *L. delbrueckii* subsp. *bulgaricus* and *S. thermophiles*, which is four more than fructooligosaccharides and four less than galactooligosaccharides [46].

### 2.5. Other Poly-Oligosaccharides and Combinative Prebiotic Effects

It is also advisable to consider here some less studied and used poly- and oligosaccharides, but similarly promising, in our opinion, in terms of their potential bioactive properties compared with previously discussed fructans, xylans, glucans, and galactans. Additionally, we address in the present section the synergistic effects of the aforementioned prebiotics occurring when they are administered in various combinations.

Pectin is among the most promising candidates for prebiotic applications. It is a complex heteropolysaccharide of plant origin, formed mainly from residues of galacturonic acid. Its content in plants is influenced by several factors, including of botanical and anatomical origin, as well as the age and maturity of the plant [72]. The suitability of pectin for specific applications is determined by its structural features, including molar mass, neutral sugar content, fractions of “smooth” and “hairy” areas, or the degree of methylation and acetylation, which can vary greatly from one raw material to another [73]. The same is true for pectin-based oligosaccharides, which proved to be even better prebiotic candidates than pectin itself [74,75]. The ability of pectin oligosaccharides to induce shifts in the populations of healthy bacteria was found to be similar or better than that of fructooligosaccharides [75]. Samples of pectin oligosaccharides obtained by the chemical, enzymatic, and hydrothermal treatment of citrus peel showed a significant prebiotic effect towards *L. acidophilus* and especially *B. bifidum* [76]. The selectivity of the prebiotic effect exerted by pectin oligosaccharides obtained from the pulp of sugar beet was also confirmed: fractions of oligosaccharides stimulated the growth of lactic acid bacteria without a noticeable effect on pathogenic strains of *E. coli* [77]. Another impressive example is the inducing effect exerted by pectin on the growth of Aspergillus sojae for the production of polygalacturonase via solid-state fermentation [78,79].

Chitosan, a deacetylated chitin derivative, is a linear aminopolysaccharide consisting mainly of repeating units of β- (1 → 4) 2-amino-2-deoxy-d-glucose (d-glucosamine) [80]. In terms of microbiology, chitosan and its derivatives in most cases manifest antimicrobial activity, both antibacterial and antifungal [81,82,83]. Despite this, some studies have demonstrated the positive effects of chitosan on beneficial bacteria used in biotechnology. Thus, fodder chitosan promoted the growth of *L. plantarum* and *P. acidipropionici* during the fermentation of a soybean plant [84]. Chitosan also slightly promoted the growth of *S. thermophilus* CR57, a starter culture used in cheese manufacturing during the storage of spreadable cheese [85]. Liu et al. (2018) [76] succeeded in obtaining a water-soluble chitosan derivative that significantly stimulated the growth of intestinal *Lactobacillus* and at the same time inhibited opportunistic *Enterococcus faecium* and *Parabacteroides distasonis* in experiments in vivo [86]. An equally important observation is the bacteriocinogenic activity of chitosan, which plays a key role in its selective effect on harmful and beneficial bacteria. A combination of chitosan and lactic acid bacteria was found to achieve a noticeably greater decrease in the number of pathogenic strains of *L. innocua*, *S. aureus*, *E. coli*, and *S. typhimurium*, as well as yeast and mold, than when they are used separately, which may indicate that chitosan stimulates increased bacterial production [84,87]. Studies on prebiotic potential were also carried out in relation to chitosan oligosaccharides. As mentioned above, chitosan oligosaccharides, as well as xylo-, mannano-, and fructooligosaccharides, increased the resistance of probiotic lactobacilli to thermal, chemical, and enzymatic effects [58]. Other studies indicate a greater prebiotic potential of chitooligosaccharides as compared to fructooligosaccharides, since the former ones accelerated the growth of lacto- and bifidobacteria to a greater extent [88], as well as exerting other selective effects on beneficial and pathogenic bacteria [89].

However, the negative effect of chitosan in nanopowder form on yogurt starter cultures should be noted: when supplementing more than 0.7% of nanopowdered chitosan, a significant decrease in the number of starter lactic acid bacteria was observed, which can be explained by the predominant antibacterial activity of chitosan, which is increased when it is supplied in nanoform [90].

The raffinose oligosaccharides isolated from the seeds of Lupinus albus var. Multolupa are also worth mentioning here. When added to milk, an increase in the populations of *B. lactis* and *L. acidophilus* starter cultures and a reduction in the fermentation time when obtaining a fermented milk product was observed [91].

The chemically modified dextrin obtained by heating potato starch in the presence of hydrochloric acid and tartaric acid, which has demonstrated prebiotic properties in relation to three strains of lactobacilli (*L. casei* Shirota, *L. casei* DN 114 001, *L. rhamnosus* Lakcid) and two ones of bifidobacteria (*B. animalis* DN 173 010 and B. bifidum Bb12), is impossible not to mention [92]. Litesse^®^, a brand of polydextrose, a synthetic polysaccharide made from randomly cross-linked glucose residues with various types of bonds, is used as a potential fat substitute in the production of kefir, and therefore its influence, especially on kefir’s microbiological profile, should be taken into account. Polydextrose had a negligible effect on the number of streptococci and yeasts and even enhanced the number of lactobacilli marginally [93].

A reasonably wide and comprehensive study comparing different prebiotics to the most prevalent probiotic bacteria was demonstrated by Su et al. (2007). According to their findings, *L. acidophilus* grew best in a basal medium supplemented with soy oligosaccharides, followed by fructooligosaccharides and inulin, while *B. animalis lactis* grew the fastest in a basal medium supplemented with soy oligosaccharides, followed by raffinose, fructooligosaccharides, hydrolysaccharides β-glucan and inulin; *L. casei* grew fastest in a basal medium supplemented with fructooligosaccharides, inulin, soy oligosaccharides, β-glucan hydrolyzate, and β-glucan concentrate, in that order [16].

## 3. Amino Acid Supplements

The choice of nitrogen source also has a direct impact on the growth and development of a producing microorganism, which is key for the biotechnological and food industries [94]. As previously emphasized, lactic acid and bifidobacteria are two of the most commercially significant bacteria types in the food sector. De Man, Rogosa, and Sharpe (MRS), a commercial ready-made substrate extensively used in laboratory research, is one of the most regularly applied growth media. This medium contains beef and yeast extracts and peptone as the major sources of nitrogen [95]. However, such a medium is usually insufficient for the transfer from laboratory to production scale, as it requires a significant increase in the number of microorganisms. Milk, however, which acts as a nutrient substrate for fermented milk production, lacks an adequate supply of free peptides and amino acids sufficient for optimal bacterial growth [96]. In this regard, each distinct species of lactobacilli should be considered to have specific growth demands on the primary energy sources, carbon and nitrogen [94]. Furthermore, the capacity to synthesize amino acids is limited in some lactic acid bacteria species. Consequently, they depend on exogenous sources of amino acids and peptides [97]. Therefore, selecting the correct bioavailable nitrogen-containing ingredients and additives to prepare an effective fermentation medium is also one of the most crucial steps in the production of concentrated starter cultures and their commercial use.

In this regard, the use of peptide and amino acid supplements from various sources in the form of hydrolysates, isolates, and other forms in order to boost the biological activity of beneficial strains of microorganisms is acceptable to address in this chapter. Table 2 lists the most notable of them.

### 3.1. Protein Hydrolysates

In recent years, the production of protein hydrolysates containing biologically active peptides from animal and plant sources using enzymatic hydrolysis has gotten a lot of attention due to their positive effect on stimulating the growth of probiotic bacteria [98]. Protein hydrolysates’ capacity to provide free amino acids, low-molecular-weight peptides, and growth factors may contribute to the growth stimulation of lactic acid bacteria. Amino acid composition, amino acid form (only L-stereoisomers or L-amino acids can enter the cell biomass), and peptide form (oligopeptides, di-, tripeptides, or free amino acids) have also been found to determine the biological value of a particular protein hydrolysate as a source of nitrogen in growth media [94]. Therefore, protein hydrolysates obtained from a variety of sources should be considered here.

Casein is the most researched peptide supplement for microbiological purposes since it is the primary protein component of milk, accounting for about 80% of the total protein content [99]. Various casein hydrolysates, particularly those hydrolyzed by the papain enzyme, have been shown to significantly contribute to the growth, survival, and production of lactic acid, and the synthesis of exopolysaccharides in lactic acid bacteria such as *L. delbrueckii* subsp. *bulgaricus*, *S. thermophilus*, *Lb. plantarum*, *Lb. sanfranciscensis*, *Lb. brevis*, as well as the growth of bifidobacteria and the proteolytic activity of *L. acidophilus* and *L. helveticus* [100,101,102,103,104,105,106,107,108]. Possibly, due to the smaller size of the peptide fragments (less than 3 kDa) which are more easily absorbed by bacteria, the hydrolysates produced with papain proved to be the most efficient in contrast to casein hydrolyzed by other enzymes.

According to some reports, eggs are one of the richest and most inexpensive sources of high-quality protein, as they contain large amounts of proline, arginine, and glutamine, which are essential amino acids for growth [109,110]. Indeed, one study demonstrated that the hydrolysates of egg white powder with a high content of free amino acids, including threonine and leucine, obtained by enzymatic hydrolysis and spray drying, supported the growth of a number of lactobacilli, including *L. plantarum*, *L. acidophilus*, and *L. reuteri* [111]. Egg protein hydrolysate also boosted the growth of *S. thermophilus*, *L. delbrueckii*, and *B. lactis* when mixed with other hydrolysates such as bovine whey and soy proteins [98].

Soy, as a protein-rich material, is widely recognized as a natural, functional food ingredient with exceptional nutritional value. Soy protein is difficult to digest due to its complicated molecular structure. However, enzymatically prepared soy peptides are now widely employed in the food and feed industries [112]. Hongfei et al. (2013) [113] managed to obtain soy protein hydrolysates suitable for the assimilation of *S. thermophilus* with fragment lengths ranging from two to eight amino acid residues, and such fragments had a stimulating effect on the proliferation of bacteria [113,114].

Some alternative and more economically promising sources of peptides have also been reported. For example, Meli et al. succeeded in obtaining peptide hydrolysates from poultry by-products based on keratin protein. Such peptides could support the growth and aminopeptidase activity of several species of *Lactobacillus* and *Bifidobacterium* [115,116].

### 3.2. Protein Isolates and Concentrates

Protein concentrates and isolates are a functional ingredient commonly utilized to improve the physical and structural qualities of fermented milk products. It also improves the product’s nutritional value and biological health impacts.

Such supplements are also worth noting as they have biological activity in relation to probiotic bacteria. Thus, whey protein concentrate enhanced the growth of *B. lactis* in milk, and increased the viability of *S. thermophilus*, *L. delbrueckii* subs. *Bulgaricus*, and *B. animalis* in low-fat yogurt significantly more than fructooligosaccharides [113,117]. In addition, the supplementation of this concentrate did not promote the excessive production of lactic acid by lactic acid bacteria, which positively influenced the stability of the yogurt during a 21-day storage period [118]. Whey protein concentrate, on the other hand, was found to be far more efficient in promoting lactobacilli development than bifidobacteria [119]. Whey protein isolate, which is freer of ballast components than protein concentrate, increased the biomass of kefir grains significantly more (392%) than modified whey protein and inulin (223%) [120]. Simplesse^®^ whey protein concentrate was employed as a potential fat substitute in one of the aforementioned research projects dedicated to the manufacture of defatted kefir, in addition to polydextrose, whose influence on microbial composition was studied. This protein supplement slightly reduced the level of yeast and streptococci while increasing the number of lactobacilli, thus affecting the microbial composition differently than Litesse^®^, allowing the product to have a longer shelf life [93]. Another protein-based fat substitute, Versagel^®^, significantly improved the growth of *S. thermophilus* and *B. longum* while inhibiting the growth of *L. casei*, *L. acidophilus*, and *L. delbrueckii* ssp. *bulgaricus*. It also adversely affected the proteolytic activity of all organisms except *B. longum*, although the activity of inhibiting angiotensin I-converting enzyme and inhibiting α-glucosidase increased [121].

Soy protein isolate also proved to be a bioactive supplement to boost the enzymatic activity of lactic acid bacteria starter cultures. There was a decrease in the synthesis of lactic acid by bacteria in two tests of its addition to probiotic and starter cultures in milk and yogurt, respectively, but an increase in acetic acid, which led to a decline in total acidity and, consequently, a loss in bacterial viability [122]. Supplementing yogurt with the isolate, on the other hand, improved the bacterial biotransformation of isoflavone glycosides to bioavailable isoflavone aglycones [123].

### 3.3. Other Peptide Supplements

Caseinmacropeptide, a hydrophilic glycopeptide obtained from the action of chymosin on κ-casein during the process of milk curdling in cheese production, contains not only available nitrogen for bacterial growth, but also amino sugars such as sialic acid and N-acetylgalactosamine, as described above. Whey protein concentrate, on the other hand, appeared to be able to increase the amount of *B. lactis* in milk, albeit to a lesser extent than protein concentrate [117]. Similarly, glycopeptide hydrolysates obtained via the glycation of β-lactoglobulin and sodium caseinate with galactose and lactose, and subsequently hydrolyzed by digestive enzymes, affect not all strains of potential probiotics. All of the lactobacilli and bifidobacteria tested absorbed B-lactoglobulin hydrolysates, and in most cases, bacterial growth was greater than when using tagatose. In addition, such glycoconjugates stimulated bacterial growth more efficiently than non-hydrolyzed β-lactoglobulin fractions. In contrast to glycated and degraded sodium caseinate, which equally stimulated the development of all tested bacterial strains, this supplement was not absorbed and did not increase the growth of thermophilic streptococci [124].

Lactoferrin is a naturally occurring iron-binding glycoprotein, found mostly in milk and is also secreted in most external fluids of mammals. It is considered one of the most significant components of the body’s defense system due to its antimicrobial activity [125]. However, Chen et al. (2014) suggested that bovine lactoferrin may also have prebiotic properties. As a result, they were convinced that depending on the concentration, lactoferrin reduces the growth rate of *B. bifidum*, *B. Infantis*, *B. longum*, *B. lactis*, *L. reuteri*, *L. coryniformis*, and *L. rhamnosus* ATCC 53103 to varying degrees, although at the later stages of growth, it increased this parameter for *L. rhamnosus* ATCC 7469 and *L. acidophilus* BCRC 14065 by about 40–200%, and also inhibited the growth of pathogens at least four times more than probiotics [126]. In the next study, the authors decided to take other species and strains of probiotic bacteria and slow/inhibit growth by incubating them at various temperatures. As a consequence, they found a more favorable prebiotic activity of bovine lactoferrin in relation to several probiotics whose growth was slowed down at room temperature. Thus, *B. breve*, *L. coryniformis*, *L. delbrueckii*, *L. acidophilus*, *B. angulatum*, *B. catenulatum*, and *L. paraplantarum* completely stopped growing at 22–24 °C. However, their growth resumed after the medium was supplemented with the additive, as was the growth of *Pediococcus pentosaceus*, *L. rhamnosus*, and *L. paracasei* [127].

α-lactalbumin, a protein that improves the bioavailability of calcium in milk and plays a key role in the hydrophilic properties of casein micelles, is another significant component of milk [128]. The effect of α-lactalbumin-hydrolyzate-calcium complexes on the microbiological properties of yogurt has been investigated. This supplement was discovered to effectively stimulate the growth of *S. thermophilus* and the fermentation rate at low concentrations in the yogurt production, but at high concentrations, it reduced the amount of *L. bulgaricus* [129].

In the same section, various studies on the effects of specific amino acids on microorganisms should be considered. For example, the effect of specific amino acids on one of the *L. plantarum* strain’s proteinase activity was investigated. Leucine and serine had the highest influence on this parameter compared with the arginine, lysine, alanine, and glutamic acid [130]. The combination of cysteine with tocopherols has also been noted to promote the growth of some *L. acidophilus*, *L. casei*, and *L. plantarum* strains [131]. Another study examined the effect of the arginine with malate supplementation on the capacity of *L. lactis* NCDO 2118 to produce γ-aminobutyric acid via glutamate decarboxylation. Arginine was found to be able to significantly increase GABA synthesis, and its combination with malate allows it to be produced in the early stages of strain development while also enhancing bacterial biomass growth [132].

## 4. Other Supplements

The third group of bioactive supplements should include everything that also has an effect on the above-mentioned microorganisms, according to current research. Vitamins, minerals, organic acids, and numerous multicomponent supplements (plant extracts, etc.) are examples of this. Table 3 lists the most notable of them.

### 4.1. Vitamins and Some Organic Compounds

Vitamins are necessary for microorganisms as coenzymes and functional groups of certain enzymes [133]. The influence of various B and C vitamin groups on the growth of the kefir grains’ biomass in low fat milk by Demirhan et al. is one of the most important research projects identified on the effects of vitamin addition on producing microorganisms in food biotechnology. They found that all the tested vitamins (B1, B6, B9, B12, C) inhibited the growth of kefir grains [134]. However, other authors have previously demonstrated the proliferative effect of some B vitamins (B1, B2, B3, B5, B7, B9) on the growth of several *L. acidophilus* and one *L. gasseri* strains, as well as an increase in their enzymatic activity towards soy oligosaccharides and, as a result, the assimilation of simple carbohydrates [135]. A recent study also examined the effects of ascorbic acid (vitamin C) and tocopherols (vitamin E) on lactobacilli strains’ development and viability. The combination of vitamins C and E did not increase the number of viable cells or shorten the milk fermentation time in the development of the fermented milk product. In contrast, the supplementation of vitamin E with l-cysteine boosted biomass fermentation without affecting the fermentation rate [131]. The same effect was observed when tocopherols were added with phytosterols in the case of a thermophilic streptococcus starter culture in cheese manufacturing [136].

Other authors were interested in the effect of polyphenols, bioactive compounds with antioxidant and antibacterial activities that are beneficial to the human body, on intestinal bifidobacteria. They noticed both stimulating and inhibiting dose-dependent effects on *B. adolescentis* and *B. bifidum* when adding narininin, hesperidin, rutin, and quercetin, as well as gallic, caffeic, p-coumaric, ferulic, chlorogenic, vanilla, and sinapic acids. Thus, hesperidin and quercetin markedly reduced the growth of both bifidobacteria species; however, in the first hours of incubation, rutin boosted the growth to 37%, and quercetin, hesperidin, and naringin boosted it up to about 20% in the case of *B. bifidum*. The largest stimulating effect was observed with coumaric acid supplementation, which induced an increase in growth in the earliest stages to about 50% of *B. bifidum* and had no effect on *B. adolescentis* [137].

Lactic acid bacteria are known to not only contribute to the final product’s development, but also directly extend its microbiological shelf life by producing organic acids, hydrogen peroxide, cyclic dipeptides, bacteriocins, fatty acids, carbon dioxide, ethanol and diacetyl, which inhibit the growth of spoilage and pathogenic microflora [138]. Valerio et al. (2016) studied the antifungal activity of certain strains of *L. plantarum*, *L. fermentum*, *L. brevis*, and *L. paracasei* caused by phenyllactic and polyporic acids, the precursor of which is phenylpyruvic acid. They found that in the presence of phenylpyruvic acid, these lactobacilli strains actually exhibit higher antifungal activity [139].

Carboxylic acids, being one of the main participants in metabolic processes in microbial cells, have also established themselves as biostimulating supplements. As previously stated, when arginine and malate are combined, *L. lactis* NCDO 2118 not only produces more GABA, but also does so in an earlier growth phase [132]. Moreover, the supplementation of citrate to a *Lactobacillus crustorum* LMG 23699 starter strain during fermentation promotes the production of some aromatic compounds by this lactic acid bacteria strain. These compounds impart a distinct flavor to baked goods manufactured with this strain [140].

### 4.2. Mineral Supplements

In addition to the impact of vitamin supplementation, Demirhan et al. (2013) investigated the effect of Cu^2+^, Mn^2+^, and PO4^3−^ ions on kefir grains. The addition of Na_3_PO_4_ × 12H_2_O stimulated the biomass growth of kefir grains the most, whereas CuSO_4_ × H_2_O had no effect on microorganisms growth [134]. Previously, the same authors studied the effect of MgO, MgSO_4_ × 7H_2_O, ZnSO_4_ × 7H_2_O, CaSO_4_ × 1/2H_2_O, and Fe_2_O_3_ introduction into milk with kefir grains. MgO had the greatest effect on growth, increasing the kefir grains’ biomass by 46.3%, and Fe_2_O_3_ was the only supplement that did not show any effect [141].

Furthermore, Cheng et al. (2019) [126] studied the effect of several inorganic salts on proteinase production by the *L. plantarum* strain, including Na_2_HPO_4_, NaH_2_PO_4_, K_2_HPO_4_, KH_2_PO_4_, CH_3_COONa, and C_6_H_14_N_2_O_7_. Proteinase production was found to be highest in cultures supplemented with Na_2_HPO_4_ and CH_3_COONa, while it was the lowest when supplemented with K_2_HPO_4_ [130].

Among metals, copper warranted specific attention as a bioactive microbial supplement. Rodríguez et al. (2008) [142] examined the effect of copper sulfate supplementation on the starter lactic acid bacteria and propionic acid bacteria used in the production of Emmental cheese (*L. delbrueckii*, *L. helveticus*, *L. rhamnosus*, *S. thermophilus*, and *Propionibacterium freudenreichii)* and found that copper inhibits growth of the chosen strains, particularly *S. thermophiles*. *L. delbrueckii* was discovered to be the most copper-resistant bacterium [142]. Nonetheless, in studying the effect of copper on the post-acidification of yogurt, it was possible to establish a supplement concentration at which a fermented milk product’s shelf life could be extended without diminishing the viability of *S. thermophilus* [143]. In addition to food production using lactic acid bacteria, copper has shown promise as a bioactive supplement in microbial food and sludge waste processing, where lactic acid bacteria can also play a key role. Unlike Fe^3+^, Mg^2+^, and Mn^2+^ ions, which lowered lactobacilli lactic acid production, copper ions stimulated the hydrolysis of carbohydrates and glycolysis, increasing it by 77%, and, unlike the previously mentioned streptococci, increased the number of lactobacilli themselves by 82.6% at a certain dosage [144]. On the other hand, Fe^2+^ iron ions were previously demonstrated to do not decrease lactic acid production, as well as the number of starter lactic acid bacteria when added to yogurt [145]. These differences are possibly due to the valence of iron, as in the last study it was Fe^2+^ ions.

Selenium is a trace mineral essential for living organisms to maintain physiological functions. Therefore, fortifying staple foods with selenium is an effective strategy to correct selenium insufficiency. According to some reports, greater efficiency in enriching food with selenium can be achieved by direct accumulation in microorganisms for probiotic supplements, since lactic acid bacteria and bifidobacteria are able to reduce and accumulate it from the nutrient medium [146,147,148,149]. In this regard, the usage of selenium as a supplement that affects the microorganisms themselves was not overlooked. Thus, selenization affected neither the growth nor viability of *Lactobacillus brevis* and *Fructobacillus tropaeoli* during storage and in the gastrointestinal tract [150]. However, in one of the studies, enrichments with selenium were proved to increase the antibacterial activity of lactic acid bacteria (*L. delbrueckii* ssp. *bulgaricus* and *S. thermophilus*) against pathogenic species while also reducing selenite to selenium and making it non-toxic, which improves its probiotic functioning [151].

### 4.3. Multicomponent Supplements

This category includes biostimulating supplements for microorganisms that consist of various compound complexes.

#### 4.3.1. Plant Extracts

Some plant extracts rich in biologically active compounds are among the most actively used complex food additives. Plant extracts are known to have antibacterial and antioxidant properties that are advantageous to human health [152]. The capacity of plant-derived extracts to boost the growth of starter cultures and probiotic bacteria should also be studied in this regard.

For example, one group of researchers found that adding *Cudrania tricuspidata* and *Morus alba* L. leaf extracts to yogurt resulted in a significant reduction in the fermentation time and an increase in the viability of yogurt starter cultures in comparison with a control sample. The combination of extracts of monosaccharides (glucose, fructose, lactose, galactose), formic, hydroxycinnamic, as well as non-chlorogenic, chlorogenic, and caffeic acids was found to play a stimulating role in relation to bacteria [153]. Another study found that when moringa extract was added, the number of viable lactic acid bacteria in yogurt, such as *S. thermophilus* and *L. acidophilus*, was higher than 106 CFU/mL, which is the standard value for the number of viable lactic acid bacteria in dairy products. This may be due to moringa extract’s high content of polyphenols [154]. The presence of polyphenols may also explain the increase in the amount of *S. thermophilus* and *Lactobacillus* during the 12 h of fermentation upon the supplementation of white mulberry leaf extracts to yogurt [155]. With the addition of grape cake extract, phenol concentration in fermented milk products increased considerably. Grape cake extract was discovered to have a protective impact on the viability of *L. acidophilus*. However, after 14 days of storage, the *L. acidophilus* populations were significantly lower compared to the *L. rhamnosus* populations, with only the last probiotic species retaining viability above 7 log CFU mL^−1^ throughout the research period [156]. In addition, Yin Lau et al. (2019) [157] discovered a dose-dependent effect of cranberry cake extract on starter cultures for meat fermentation. According to them, the maximum growth-promoting concentration for the genera *Lactobacillus* and *Pediococcus* was slightly higher than for *Staphylococcus* spp., whereas extract concentrations above a certain value inhibited their growth [157].

The observations made by Michael et al. (2015) [158] are also noteworthy. They found that yogurts supplemented with a commercial plant extract (consisting of olive, garlic, onion and citrus extracts) retained a greater amounts of *L. bulgaricus* and *L. acidophilus* at the end of storage than yogurts without supplements, but these extracts had no significant effect on *S. thermophilus* and *B. animalis* [158]. When *Siraitia grosvenorii* fruit extract was added to probiotic yogurt, a similar impact was detected, with *L. bulgaricus* ranging from 8.16 log CFU/g to 8.83 log CFU/g, and they tended to be slightly lower than *S. thermophilus* in all yogurt samples. Although the supplementation of 0.5% extract did not significantly affect the amount of *L. bulgaricus*, 1% and 2% of the extract significantly increased bacterial viability. Moreover, the supplementation of this extract significantly improved the viability of *L. casei* compared to the control probiotic yogurt. An aqueous extract of *S. grosvenorii* was found to contain soluble fiber, monosaccharides, essential amino acids, and flavonoids, which can provide a prebiotic effect for *L. bulgaricus* and *L. casei* [159].

Infusions of 22 South African traditional leafy plants stimulated at least one of the four probiotics studied (*L. bulgaricus*, *L. lactis*, *L. reuteri*, and *B. longum*) in pure culture due to their high inulin content (from 2.5% to 3.6%). *Sonchus oleraceus* stimulated all four strains and *Taraxacum officinale* stimulated three. A total of 18 plants stimulated at least one of the four probiotic strains [160].

Tea has become a rather interesting and demanded object, with promise as a biostimulating supplement. The phenolic substances present in tea are known to be capable of modifying the intestinal microbiota, inhibiting the growth of pathogenic bacteria, and increasing the level of commensal bacteria, including bifidobacteria, which indicates their prebiotic effect [161]. Tea extract was observed to significantly stimulate the reproduction and acidification of probiotic strains such as *L. rhamnosus* GG, *L. acidophilus* NCFM, and *L. plantarum* ST-III TE, but did not significantly affect *B. bifidum* Bb02. Thus, its supplementation to skimmed milk was beneficial for lactic acid bacteria fermentation, but not for their growth rate [162]. Pu-erh tea also slightly improved the viability of *L. acidophilus* and lowered the pH of probiotic yogurts [163]. Simultaneously, the inclusion of the green tea infusion effect in bioyogurt on the survivability of *L. acidophilus* LA-5 depended on the dose. Thus, the higher the infusion dosage, the lower the number of these bacteria was found in the fermented milk product. The supplementation of 5% green tea resulted in significantly more lactobacilli (almost 1 log) than higher tea dosages [164].

Marine macroalgae are one of the richest and most promising sources of bioactive primary and secondary metabolites that can be extracted and have also found their place in the field of application we are interested in [165]. However, different levels of *Gracilaria domingensis* aqueous extract did not affect the product fermentation process. The profiles of titratable acidity, pH value, and the size of microbial populations of *S. thermophilus*, *L. acidophilus* and *B. animalis* ssp. *lactis* were the same for all samples during the fermentation [166]. Among the positive effects of plant extracts, it is worth noting how the addition of rice extract to yogurt greatly stimulated the growth of not only lactic acid bacteria, but also bifidobacteria, which was probably due to its high content of panose [167]. Despite the predominantly antimicrobial properties of *Aloe barbadensis* and *Aloe arborescens* [168], aqueous extracts of the internal parenchyma did not inhibit the growth of lactic acid bacteria, and the growth stimulating effect on *L. acidophilus* was observed at 5% inclusion [169].

#### 4.3.2. Plants, Their Components, and By-Products

Numerous studies have shown that plant products, particularly those obtained from fruit processing (peel, cake, seeds), are good sources of dietary fiber and other biologically active compounds [170,171,172]. For example, pineapple peel and cake powders were rich in nutrients such as dietary fiber, protein and bivalent cations, and demonstrated a prebiotic effect on *L. acidophilus*, *L. casei*, and *L. paracasei* spp. *paracasei*, and the supplementation of persimmon leaf powder to yogurt increased the amount of *S. thermophilus* and *Lactobacillus* starter cultures during 12 h of fermentation [155,173].

The supplementation of passion fruit by-product to mMRS broth with cultures of *B. longum* subsp. *infantis* BB-02 and *Lb. reuteri* RC-14 had an interesting effect, increasing folic acid synthesis after 24 h of fermentation [174]. However, passionfruit peel powder did not cause any significant change in the number of probiotics, with the exception of *B. lactis* Bl04 in whole yogurt, which was 0.8 logs higher than in the control group. Perhaps, taking the entire storage period into account, the average titratable acidity in yogurts containing passion fruit peel powder was noted to be significantly higher than in their respective controls [175]. Passion fruit fiber was also noted to increase conjugated linoleic acid in all probiotic yogurts, and all tested fruit fibers overcame the negative effect of *L. acidophilus* L10 on conjugated linoleic acid in yogurts. Furthermore, because of their high levels of phenolic compounds, carotenoids, and buffering fibers, passion fruit, guava, and orange by-products contributed to the growth of *L. casei* Lc-1 and *S. thermophilus* TA040 during fermentation, as well as maintaining their population when storing fermented drinks based on goat milk, as well as oats and rice [176]. At the same time, as with pineapple peel powder, the supplementation of passion fruit peel powder to probiotic yogurt did not significantly affect the quantity of *S. thermophilus* [177]. However, the supplementation of banana fiber in the same study significantly increased the content of α-linolenic acid in yogurt [177].

The interesting effects of plant fiber supplementation have been observed by Sendra et al. (2008) [178]. Citrus fibers increased the survival of *L. acidophilus* CECT 903 and *L. casei* CECT 475 in MRS broth when stored in a refrigerator, but the results obtained for *B. bifidum* CECT 870 were uneven. Fibers from orange increased their growth, while fibers from lemon had an inhibitory effect [178]. In turn, apple and banana fiber helped to sustain the viability of all probiotic strains tested in studies for the production of fiber-fortified low-fat yogurts, notably *S. thermophilus*, *B. animalis* subsp. *lactis* Bl04, HN019, and B94, and *L. acidophilus* L10 for up to four weeks cold storage. All fiber-enriched yogurts had greater levels of short-chain fatty acids and polyunsaturated fatty acids than the control group. A synergistic effect has been noted between the fiber type and the probiotic strain at the level of conjugated linoleic acid [177].

*Oliveria decumbens* Vent. has the proper antibacterial effect due to the rich presence of essential oils [179]. Despite this, dried flowers added to the plant increased the growth rate of *L. acidophilus* and *B. bifidum* in milk and yogurt during storage and helped to achieve the desired acidity in a shorter period [180]. The latter property was similarly influenced by the supplementation of sprouted soy seeds to the nutrient medium to a sourdough consisting of such *lactobacillus* strains as *Lb. plantarum* LOCK-0860, *Lb. sanfranciscensis* DSM-20663, and *Lb. brevis* DSM-1267, which is clearly explained by their high nutritional value [107].

Aloe vera powder supplementation (1%) to skim milk resulted in a significant increase in the ACE inhibitory ability to fermented milk. The increase in the ACE inhibitory activity with the aloe vera supplementation corresponded to a change in the proteolysis degree during milk fermentation by the probiotic microorganisms. The supplementation of aloe vera powder also increased the viable amounts of *L. casei* NCDC 19 in fermented milk during storage for 7 days, and these values remained quite high [181].

In vitro studies on the growth of selected probiotics using different plant spices have shown that this type of nutritional supplement significantly boosts probiotic growth while suppressing pathogens [182]. For example, yogurt with cardamom added has shown a marked increase in *Bifidobacterium* during storage [183].

*Spirulina* sp. is the most well-known genus of algae used in food due to its nutritional value. It contains 18 of the 20 known amino acids, high quality proteins, more calcium than milk does, more vitamin B12 than bovine liver does, vitamins A, B2, B6, E, H, and K, as well as all the necessary minerals, trace elements, and enzymes [184]. The supplementation of *Spirulina platensis* biomass to fermented milk had a beneficial effect on the survival of *S. thermophilus* and *Bifidobacterium* spp., *L. delbruekii* subsp. *Bulgaricus*, and especially *L. lactis* subsp. *lactis* and *L. acidophilus*, regardless of storage temperature [185,186]. Moreover, *B. animalis* was able to sustain higher populations than control samples during the 21 days of storage of probiotic dairy desserts in the presence of *G. birdiae* or *G. domingensis* algae dispersions, although *L. acidophilus* demonstrated reduced viability in the product [187].

For plant-based processed foods, supplementation up to 3% of quinoa flour to fermented milk did not affect the fermentation kinetics or probiotic activity of *S. thermophilus*, *B. animalis* ssp. *lactis* BB-12, and *L. acidophilus* La-5 within a 28-day storage period [188]. The supplementation of rice bran to the probiotic yogurt showed a significant increase in the amounts of *L. casei* 431 compared to a control yogurt [189]. When strawberry marmalade was added to yogurt, the survival rate of *L. acidophilus* was higher than that of *B. bifidum*. However, while the viability of *L. acidophilus* decreased during the storage period, the amount of *B. bifidum* remained stable during the entire storage period of the probiotic yogurt [190].

Grapes and their byproducts are frequently employed as natural sources of antioxidants due to their high content of phenols, with special emphasis on anthocyanin and resveratrol [191]. However, the supplementation of Isabella grapes (*Vitis labrusca* L.) did not significantly affect *S. thermophiles*, *L. bulgaricus*, or *L. acidophilus* in a goat milk probiotic yogurt [192]. Plant preparations (carrots, pumpkin, broccoli, and red bell peppers) were supplemented to processed cow’s milk fermented with DVS yogurt culture. Only the product with added pumpkin pomace showed lower lactobacilli levels and a slightly higher count of streptococci than other yogurts [193]. Moreover, despite its high polysaccharide and polyphenol content [194], the pulp of ziziphus (*Zízíphus jujúba*), when added to goat milk yogurt at concentrations of 3%, 6%, and 9%, significantly reduced the amount of *S. thermophilus* [195].

#### 4.3.3. Others

*L. acidophilus* 4461, *L. acidophilus* 4962, *L. casei* 290, and *L. casei* 2607 have been used to ferment soymilk. After 24 h of incubation, the addition of skimmed milk powder significantly increased the growth of *Lactobacillus* spp., providing lactose and other nutrients, and thus playing a key role in lowering the pH of the product, as well as significantly increasing the biotransformation of isoflavone glycosides to isoflavone aglycones [196].

Apar et al. (2017) [197] evaluated the effect of yeast extract on kefir grain growth and found the greatest (1.657%) increase in the biomass of kefir grains in whey over a 10-day period when this extract was added, compared to samples with the supplementation of yogurt (1.458%) and milk (1.294%) [197].

Honey is produced worldwide by over 500 species of bees belonging to 32 genera [198], and naturally contains a large number of antioxidants (including flavonoids, phenolic and carotenoids), organic acids, Maillard reaction products, and amino acids in their composition [199], as well as a specific profile and acidity of sugar, which gives the product unique sensory characteristics [200]. The positive effect of this multicomponent product on microbiological processes in fermented milk product manufacturing would be quite fair. On the one hand, honey supplementation ranging from 1.0% to 5.0% (*w*/*v*) was found to have no significant effect on the survival of *S. thermophilus* and *L. delbrueckii* subsp. *bulgaricus* in yogurt for 6 weeks at 4 °C. Similarly, honey had no effect on pH and lactic acid levels in the final products [201]. However, when sunflower honey was added to the samples, the number of *S. thermophilus* and *L. delbrueckii* subsp. *bulgaricus* rose dramatically in contrast to the control group samples, as did their vitality [202]. Goat milk yogurt containing *Melipona scutellaris* honey showed the highest amount of *L. acidophilus* La-05, at about 1 log CFU/g, indicating a growth stimulating effect [203]. The growth kinetics of *P. pentosaceus* and *L. fermentum* starter cultures used in baking was positively influenced by the supplementation of *Prosopis* sp. honey in the amount of 6.5% (*w*/*v*). Despite the fact that the population size was the same after 19 h of fermentation, *P. pentosaceus* had a stronger effect on the acidification process than *L. fermentum*. The latter, on the other hand, showed a higher growth rate than *P. pentosaceus* [204].

## 5. Conclusions

Taken together, all the above observations have shown that the effect of boosting the growth of beneficial microorganisms and their various metabolic activities related to food biotechnology in fermented and/or probiotic food products depends both on the supplement used and on the bacterial species/strain targeted. The efficiency of these supplements is determined not only by the content of the essential substances therein, but also by their bioavailability with regard to specific species/strains of bacteria, as well as the level of demand for these compounds. This conclusion can be derived from a systematic analysis taking into account the exact type of the nutrient and set of biologically active compounds it contains. Mainly, this review should have shed light onto issues of advisability of using certain compounds as additives to growth media during the cultivation of microorganisms in order to obtain fermented food products, and then help in determining which supplements would be the most complete and affordable source of all the essential components. According to our analysis, the main emphasis in contemporary studies is placed onto economic aspects, to find the most cost-effective and accessible sources. In addition, some of the considered supplements were incorporated into a product not to stimulate microbial processes, but rather to improve on other product features, i.e., to directly influence the sensory (taste/flavor) and physicochemical properties of the final product. However, their effect on the microbiological profile of the product has been studied as well, to guarantee that such supplements, at worst, have no effect on beneficial microorganisms in the product, such as probiotics. The issue of the ambiguity and even unpredictability of the effects of such bio-additives on microorganisms is a key limitation to their widespread involvement in microbiological food production. It is therefore reasonable to see a wider and more comprehensive study of them as a near-term goal. In summary, we have managed to perform a complete and almost comprehensive review of the results obtained to date concerning the effects of various supplements, both single- and multi-component ones, on the microorganisms widely applied in fermented dairy products development.

## Figures and Tables

**Table 1:** The most notable poly-/oligosaccharide bioactive supplements.

Biologically Active Compound	Substrate/Food	Microorganisms	Effect/Functionality
Inulin	Reconstituted skim milk	*S. thermophilus*, *Bifidobacterium longum*	Improving growth
	Soymilk	*Lactococcus*, *Lactobacillus* and yeasts	Increasing the survival rate
	Yogurt	*S. thermophilus*, *L. acidophilus* and *Bifidobacterium* sp.	Improving growth
Branched fructans		*L. plantarum*, *L. casei*, *Lactobacillus fermentum*, *B. catenalatum*, *B. bifidum*, *B. longum*, *B. animalis* spp. *lactis*	Growth stimulation
Fructans *Agave salmiana*		*L. acidophilus* and *B. lactis*	Prebiotic effect
Fructans of *A. salmiana*		*L. casei* and *B. lactis*	Prebiotic effect
Fructooligosaccharides	Yogurt	*S. thermophilus*, *L. acidophilus* and *Bifidobacterium* sp.	Improving growth
	Fermented soy milk		Metabolism improvement
	Soymilk	*S. thermophilus* TH-4, *L. acidophilus* LA-5, *L. rhamnosus* LGG, *L. fermentum* PCC and *L. reuteri* RC-14	Raise activity of folic acids and increasing the survival rate
Combination of fructooligosaccharides and galactooligosaccharides		*L. plantarum* ZLP001	Vitality improvement with heat stress
Fructooligosaccharides and soy oligosaccharides			Vitality improvement with cold stress
Oligofructose	Ice cream mix	*L. acidophilus* La-5 and *B. animalis* Bb-12	
Lactulose	Soymilk	*B. lactis* and *B. longum*	Increasing the number of viable cells and enhancing biotransformation of isoflavone glycosides to isoflavone aglycones
	Yogurt	*S. thermophilus* and *L. delbrueckii* subsp. *bulgaricus*	Stimulation of growth and enzymatic processes
Galacto; trisaccharides synthesized from lactulose and lactose		*Lactobacillus*, *Streptococcus* and *Bifidobacterium*	Growth promotion
Galactan oligosaccharides		*L. delbrueckii* subsp. *bulgaricus* and *S. thermophiles*	
Xylooligosaccharides		*L. plantarum* and *L. acidophilus*	Increasing stability to heat, exposure to phenol solutions and artificial gastrointestinal juices
		*L. bulgaricus* and *S. thermophilus*	Prebiotic effect
Arabinoxylans		*Bifidobacterium breve* and *L. reuteri*	Growth-stimulating effect
β-glucan	Yogurt	*Bifidobacterium animalis* ssp. *lactis*	Improvement in viability and stability under cold stress; increasing the production of lactic and propionic acids
		Bifidobacteria	Increasing stability under cold stress
		*B. longum*	
2-substituted (1-3)-β-d- glucan		Bifidobacteria, *L. plantarum* and *L. acidophilus*	Growth-stimulating effect
Pullulan	Yogurt	Lactobacilli and bifidobacteria	Growth-stimulating and protective effect, improvement in enzymatic characteristics
β-glucooligosaccharides		*L. lactis* subsp. *lactis*, *L. reuteri*, and *Pediococcus acidilactici*	Regulate selective growth and exert antimicrobial activity
Glucooligosaccharides	Yogurt	*L. delbrueckii* subsp. *bulgaricus* and *S. thermophiles*	Growth-stimulating effect
Pectin oligosaccharides		*L. acidophilus* and *B. bifidum*	Growth-stimulating effect
Chitosan	Soybean plant	*L. plantarum* and *P. acidipropionici*	Growth-stimulating effect
	Cheese	*S. thermophilus* CR57	Growth-stimulating effect
		Intestinal lactobacilli	Growth-stimulating effect
Chitosan oligosaccharides		Probiotic lactobacilli	Increasing the resistance to thermal, chemical, and enzymatic effects
		Lacto bacilli and bifidobacteria	Growth-stimulating effect
Raffinose oligosaccharides	Milk	*B. lactis* and *L. acidophilus*	Growth-stimulating and effect, improvement of enzymatic characteristics
Chemically modified dextrin		*L. casei* Shirota, *L. casei* DN 114 001, *L. rhamnosus,* Lakcid *B. animalis* DN 173 010 and *B. bifidum* Bb12	Prebiotic effect

**Table 2:** The most notable amino acid bioactive supplements.

Biologically Active Compound	Substrate/Food	Microorganisms	Effect/Functionality
Casein hydrolysates	Yogurt	*L. delbrueckii* subsp. *bulgaricus*, *S. thermophilus*, *Lb. plantarum*, *Lb. sanfranciscensis*, *Lb. brevis*, *L. acidophilus* and *L. helveticus*	Positive contribution to the growth, survival, and production of lactic acid, and synthesis of exopoly-saccharides
		*L. acidophilus* and *L. helveticus*	Proteolytic activity
Hydrolysates of egg white powder		*L. plantarum*, *L. acidophilus*, *L. reuteri*, *S. thermophilus*, *L. delbrueckii*, and *B. lactis*	Growth-stimulating effect
Keratin hydrolysates		*Lactobacilli* and *Bifidobacteria*	Support growth and aminopeptidase activity
Whey protein concentrate	Milk	*B. lactis*	Growth-stimulating effect
	Low-fat yogurt	*S. thermophilus*, *L. delbrueckii* subs. *bulgaricus* and *B. animalis*	Increasing viability
Whey protein isolate	Kefir/milk	Kefir grains	Growth-stimulating effect
Modified whey protein	Kefir/milk	Kefir grains	Growth-stimulating effect
Simplesse^®^ whey protein concentrate	Kefir	Lactobacilli	Growth-stimulating effect
Versagel^®^ proteolytic activity		*S. thermophilus* and *B. longum*	Growth-stimulating effect
Hydrolysates β-lactoglobulin and sodium caseinate		Lactobacilli, bifidobacteria, thermophilic streptococci	Growth-stimulating effect
Lactoferrin		*L. rhamnosus* ATCC 7469, *L. acidophilus* BCRC, *B. breve*, *L. coryniformis*, *L. delbrueckii*, *L. acidophilus*, *B. angulatum*, *B. catenulatum*, *L. paraplantarum* *Pediococcus pentosaceus*, *L. rhamnosus*, *L. paracasei,*	Growth-stimulating effect
α-lactalbumin-hydrolyzate-calcium complexes	Yogurt	*S. thermophilus*	Growth-stimulating effect
Leucine and serine		*L. plantarum*	Increasing proteinase activity
Cysteine and tocopherols		*L. acidophilus* *L. casei* *L. plantarum*	Growth-stimulating effect
Arginine		*L. lactis* NCDO 2118	Increasing GABA synthesis; Growth-stimulating effect

**Table 3:** The most notable other bioactive supplements.

Biologically Active Compound	Substrate/Food	Microorganisms	Effect/Functionality
B1, B2, B3, B5, B7, B9 vitamins	Milk/kefir	*L. acidophilus* *L. gasseri*	Growth-stimulating effect; increasing enzymatic activity
Vitamin E and l-cysteine	Milk	*Lactobacillus Acidophilus* NRRL B-4495, *Lactobacillus Casei* NRRL B-1922 and *Lactobacillus Plantarum* NRRL B-4496	Boosting biomass fermentation
Tocopherols and phytosterols	Milk/cheese	Thermophilic streptococcus	Boosting biomass fermentation
Polyphenols		Coumaric acid *B. bifidum*	Growth-stimulating effect
Phenylpyruvic acid		*L. plantarum*, *L. fermentum*, *L. brevis*, and *L. paracasei*	Increasing antifungal activity
Na_3_PO_4_ × 12H_2_O	Milk/kefir	Kefir grains	Growth-stimulating effect
MgO	Milk/kefir	Kefir grains	Growth-stimulating effect
Na_2_HPO_4_ and CH_3_COONa		*L. plantarum*	Increasing proteinase production
Copper ions		Lactobacilli	Growth-stimulating effect; increasing the hydrolysis of carbohydrates and glycolysis
Selenium		*L. delbrueckii* ssp. *bulgaricus* and *S. thermophilus*	Increasing antibacterial activity
*Cudrania tricuspidata* and *Morus alba* L. leaf extracts	Milk/yogurt	*L. delbrueckii* ssp. *bulgaricus* and *S. thermophilus*	Increasing fermentation activity
*Sonchus oleraceus* infusion		*L. bulgaricus*, *L. lactis*, *L. reuteri* and *B. longum*	Growth-stimulating effect
Tea extract	Skim milk	*L. rhamnosus* GG, *L. acidophilus* NCFM and *L. plantarum* ST-III TE	Growth-stimulating effect; increasing acidification activity
Rice extract	Yogurt	Lactic acid bacteria and bifidobacteria	Growth-stimulating effect
Persimmon leaf powder	Yogurt	*S. thermophilus* and *Lactobacillus*	Growth-stimulating effect
Orange fibers	Yogurt	*L. acidophilus* CECT 903 and *L. casei* CECT 475	Growth-stimulating effect
*Oliveria decumbens* Vent. flowers	Milk/yogurt	*L. acidophilus* and *B. bifidum*	Increasing fermentation activity
*Spirulina platensis*	Fermented milk	*S. thermophilus* and *Bifidobacterium* spp., *L. delbruekii* subsp. *Bulgaricus* and *L. lactis* subsp. *lactis* and *L. acidophilus*	Increasing stability under cold stress
Rice bran	Yogurt	*L. casei* 431	Growth-stimulating effect
Skimmed milk powder	Soymilk	*Lactobacillus* spp.	Growth-stimulating effect
Sunflower honey	Yogurt	*S. thermophilus* and *L. delbrueckii* subsp. *bulgaricus*	Growth-stimulating effect; Increasing viability
